# Ce3Light: Design, Construction, and Testing of a Light-Irradiation System for In Vitro Cell Studies

**DOI:** 10.3390/bioengineering13070841

**Published:** 2026-07-21

**Authors:** Jiří Handl, Helena Radochlibová, Jan Čapek, Tomáš Roušar, Petra Babicová, Norbert Ferenčík, Mária Danko, Radovan Hudák

**Affiliations:** 1 Department of Biological and Biochemical Sciences, Faculty of Chemical Technology, University of Pardubice, 532 10 Pardubice, Czech Republic; jiri.handl@upce.cz (J.H.); helena.radochlibova@student.upce.cz (H.R.); jan.capek7@upce.cz (J.Č.); tomas.rousar@upce.cz (T.R.); 2 Department of Biomedical Engineering and Measurement, Faculty of Mechanical Engineering, Technical University of Kosice, 042 00 Kosice, Slovakia; norbert.ferencik@tuke.sk (N.F.); maria.danko@tuke.sk (M.D.); radovan.hudak@tuke.sk (R.H.)

**Keywords:** photobiomodulation, cell cultivation in vitro, LED irradiation

## Abstract

Photobiomodulation (PBM) has gained increasing attention in tissue engineering and cell biology, yet reproducible in vitro studies remain limited by the availability of standardized irradiation platforms. This study presents the design, construction, and technical validation of Ce3Light, a modular LED-based irradiation system developed for controlled photobiomodulation experiments under standard cell culture conditions. The platform was fabricated using additive manufacturing (PA12) and incorporates interchangeable LED modules, integrated monitoring of illuminance and temperature, and compatibility with conventional CO_2_ incubators. To demonstrate its applicability for in vitro research, the system was evaluated using MRC-5 fibroblasts exposed to four wavelengths (460, 530, 660, and 800 nm). Cellular responses were assessed by measuring intracellular glutathione levels and dehydrogenase activity. The experiments confirmed that the platform enabled stable and reproducible irradiation while detecting wavelength-dependent cellular responses consistent with previous reports in the literature. These findings primarily validate the functionality and suitability of Ce3Light as an experimental platform rather than establish new biological mechanisms. The developed system provides a versatile, reproducible, and adaptable tool for future photobiomodulation studies requiring precise control of irradiation conditions.

## 1. Introduction

Photobiomodulation (PBM), previously referred to as low-level laser therapy (LLLT), has become an increasingly studied topic in regenerative medicine and cell biology [1]. PBM is based on the application of low-intensity light, most commonly within the visible and near-infrared spectrum, to stimulate cellular processes through non-thermal photochemical reactions [1,2,3,4]. At the cellular level, these effects are mainly linked to the absorption of photons by cytochrome c oxidase in the mitochondrial respiratory chain, which can subsequently influence ATP production, reactive oxygen species formation, and intracellular signaling pathways [1,2,5]. There are more than sixty other names for the treatment known as “PBM therapy (PBMT),” such as “photobioactivation,” “photobiostimulation,” “LED phototherapy,” “low-level light therapy (LLLT),” “cold laser therapy,” “soft laser,” and “narrow-band light.” [3] The term “PBM” is preferred for a number of good reasons: it implies that the therapeutic benefits come from both photosignal transmission and, in some cases, inhibitory effects. Moreover, it implies that therapeutic advantages can be obtained without a laser. PBMs can use coherent light sources like lasers, incoherent light sources like LED lights, or a mix of the two [2,4,5,6].

The biological effects of PBM are strongly dependent on irradiation parameters such as wavelength, irradiance, exposure time, and total delivered dose [6]. Previous studies have shown that insufficient stimulation may produce little or no effect, whereas excessively high doses can inhibit cellular activity or even induce cytotoxic responses. This biphasic behavior is commonly described by the Arndt–Schulz law [1,2,7,8]. Fibroblasts are among the most frequently used cell models in PBM research because of their important role in tissue regeneration and wound healing, particularly in studies focused on cellular metabolism and oxidative stress [6,7,8,9,10,11,12].

Photobiomodulation has been extensively investigated in various cellular models, particularly in stem cells and fibroblasts, where light stimulation has been associated with changes in proliferation, differentiation, migration and cellular metabolism. Several studies demonstrated that red and near-infrared wavelengths can positively influence cell proliferation and tissue regeneration processes. Increased proliferation was reported in human gingival fibroblasts (HGF) following laser irradiation at 809 nm and 830 nm [13,14]. Similarly, irradiation of HGF using wavelengths between 670 and 786 nm resulted in increased fibroblast proliferation [15]. Human embryonic stem cell-derived mesenchymal stem cells (hESC-MSCs) exposed to 635 nm laser irradiation exhibited enhanced proliferation, migration and osteogenic differentiation at lower doses, whereas higher doses produced inhibitory effects [16]. Similar biphasic behaviour was observed in mesenchymal stem cells and melanoma cells irradiated at 835 nm, where low doses stimulated proliferation while higher doses induced growth inhibition and cytotoxicity [16,17].

Several studies highlighted the wavelength-dependent nature of the cellular response. Wang et al. demonstrated that irradiation of human adipose-derived stem cells (hASCs) with red (660 nm) and near-infrared (810 nm) light stimulated proliferation, whereas blue (415–420 nm) and green (540 nm) wavelengths inhibited proliferation and increased intracellular ROS production and Ca^2+^ signaling [18,19]. Interestingly, despite their inhibitory effect on proliferation, blue and green wavelengths promoted osteogenic differentiation more effectively than red or near-infrared light, suggesting that different spectral regions may activate distinct intracellular pathways.

The influence of PBM on oxidative stress regulation has also been extensively investigated. LED irradiation of L929 fibroblasts at 660 nm and 850 nm provided protection against H_2_O_2_-induced oxidative stress, increased mitochondrial activity and reduced ROS production [20]. Likewise, human dermal fibroblasts exposed to wavelengths ranging from 440 nm to 900 nm exhibited the strongest mitochondrial response at 645 nm, accompanied by enhanced resistance to oxidative stress [21].

Red and near-infrared wavelengths are generally associated with enhanced proliferation and metabolic activity, whereas shorter wavelengths often induce oxidative signaling, differentiation and antiproliferative effects [18]. At the same time, many studies consistently demonstrate a biphasic dose–response relationship, where lower doses stimulate biological activity while excessive irradiation leads to inhibitory or cytotoxic outcomes, in accordance with the Arndt–Schulz principle [16,17,22,23].

The aim of this work was to validate the technical functionality of the Ce3Light device and to evaluate its applicability in photobiomodulation experiments using the human fibroblast cell line MRC-5. The MRC-5 cell line represents a suitable experimental model primarily due to its morphological similarity to skin fibroblasts. Another advantage of this cell line is that it is derived from healthy lung tissue, therefore, its metabolism is not affected by malignant transformation or immortalization, which are common limitations of many established cell lines. As a result, MRC-5 cells provide a more physiological response to experimental interventions and stimulation [24,25]. The MRC-5 cell line has also been employed in similar photobiomodulation studies, with Jones et al. [25], for example, using it as an experimental cell model.

The system was constructed using additive manufacturing methods to provide a cost-effective and adaptable solution that allows integration of different LED modules together with real-time monitoring of temperature and illuminance during experiments. Cellular responses to four different wavelengths (460, 530, 660, and 800 nm) were analyzed.

## 2. Materials and Methods

### 2.1. Design of Ce3Light

Despite the predominance of laser systems in previous research, we decided to apply LED technology (Light Emitting Diodes). Several key factors led us to this decision, primarily the effort to achieve more homogeneous irradiation of a larger area without the difficult expansion of a special laser beam. In addition, LEDs represent a more economically accessible and operationally efficient alternative.

Our irradiation system represents an experimental platform designed for controlled irradiation of cell cultures and generation of defined light conditions in an in vitro environment. The device was developed in response to current needs in the fields of tissue engineering, biomaterial development and photobiology, where precise control of light parameters is key when monitoring cell proliferation, differentiation and metabolic activity. In addition to stimulating biological processes, the system is also optimized for controlled future photopolymerization (curing) processes of medical hydrogels prepared by 3D bioprinting or casting methods.

The structure itself consists of a closed light chamber, which can be easily placed in a CO_2_ incubator, but also operated separately. The illumination of the 96-well culture plate is realized from below using replaceable LED modules that emit light with specific parameters. The system of exchange cards, which are simply connected to the bottom, allows for an operational change of the light spectrum.

Above the light source is a variable holder for a plate with cells, which can be fixed at one of five preset heights (from 20 mm to 80 mm). This function allows us to change each adjustment of the incident radiation through the distance from the source. The entire process takes place in a closed box, which protects the surroundings from unwanted radiation and ensures light stability inside.

The intelligent side of the device consists of integrated sensors—a thermometer and a luxmeter. They continuously measure the internal microclimate and the intensity of the lighting, while storing the obtained data for later analysis. The entire box is controlled by a microcomputer connected to a tablet on top of the device. Thus, we can easily configure the exposure duration, measurement in lux or the type of source used and at the same time control the progress of the experiment in real time.

**Figure 1:**
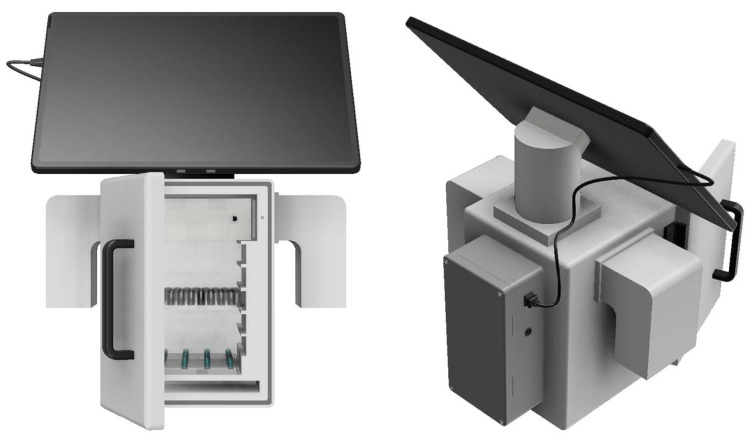
Ce3Light device model—open (Autodesk Fusion 360).

A complete 3D model of the device was developed in SolidWorks 2025 SP5.0 (Dassault Systèmes, Vélizy-Villacoublay, France), which allowed for precise dimensioning of internal components and ensuring mechanical compatibility of individual parts. Subsequently, the models were processed and rendered in Autodesk Fusion 360 (San Rafael, CA, USA) to achieve final visualization and technical preparation for production (Figure 1).

**Figure 2:**
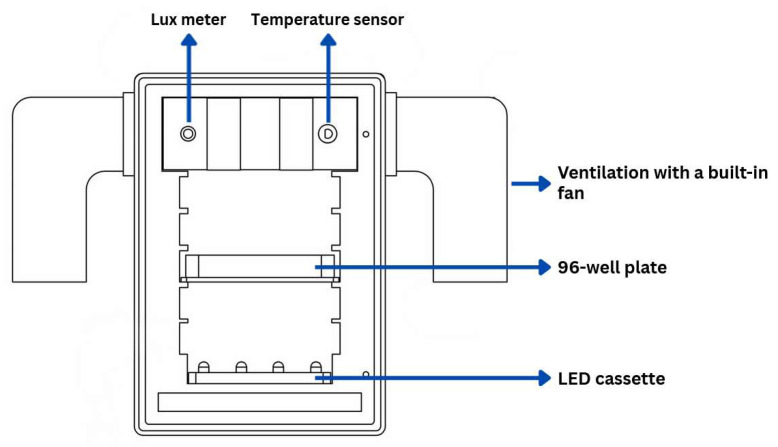
Ce3Light device configuration.

The design of the device is based on the requirement for a high degree of flexibility and adaptability under different experimental conditions. We focused on the modularity of individual components so that the device can be easily adapted to different types of samples, experimental protocols or user requirements. An important element are interchangeable LED cards, which allows for quick and easy replacement of light sources according to the desired wavelength or intensity of illumination. Device configuration can be seen in Figure 2. And device measurements in Table 1.

**Figure 3:**
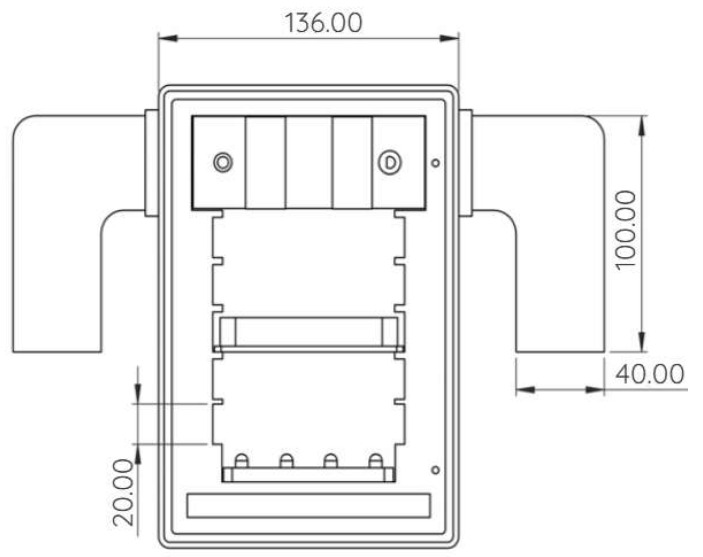
Ce3Light: Front view of 5 irradiation levels with 96-well plate.

Another important element of the design is the variable placement of samples in five height levels, which can be seen in Figure 3. This system allows us to adjust the distance of the samples from the light source, thereby increasing the variability of the experiment and the accuracy of the conditions. The height levels are designed to allow easy manipulation of the samples while ensuring their stable storage throughout the experiment.

We also took into account the need to maintain a stable internal temperature of the device. Temperature stability is particularly important in biological or biochemical experiments, where even small fluctuations can affect the measurement results. For this reason, ventilation holes are integrated into the design to allow natural air flow.

The design also allows for the use of the device in a CO_2_ incubator environment, where there are specific conditions such as increased humidity, stable temperature and controlled carbon dioxide concentration. The design solution therefore minimizes elements that could disrupt the stability of the incubator environment, while supporting natural air circulation without the need for active cooling or ventilation.

The entire system is designed as an autonomous unit, in which the hardware part is directly connected to the control software implemented in a touch tablet. This connection allows the user to easily set the experiment parameters, control the lighting process or change the device settings through an intuitive user interface. The integration of hardware and software also increases the reliability of the system and allows for easy operation.

### 2.2. Construction of Ce3Light

The choice of materials, manufacturing technology and design strategy for biomedical 3D printing must be tailored to the intended application [26,27,28,29]. In this work, the device was fabricated using selective laser sintering (SLS). The process involves uniformly depositing thin layers of PA12 powder over the entire platform, followed by selective laser sintering according to a digital design. Localized thermal energy from the laser induces partial melting and interparticle bonding, with subsequent crystallization occurring during controlled cooling. This layer-by-layer sequence is repeated until the final three-dimensional structure is completed [30,31,32].

To print the device parts, we used an EOS P390 3D printer (EOS GmbH, Krailling, Germany) from polyamide 12 (PA12). The EOS P360 has a build volume of 340 mm × 340 mm × 620 mm, with an extra-large Z-axis height of 620 mm enabling efficient vertical stacking of parts, known as nesting. The surrounding unspent powder acts as a natural support, allowing the full nominal chamber volume to be utilized without the need for additional support structures. This maximizes throughput and enables the production of large components or large batches of smaller parts in a single print run [0]. Due to the relatively large volume of the EOS P390 system, SLS enables the direct production of complex components and functional assemblies, including structures with integrated moving parts, minimizing the need for fasteners or post-assembly steps [33].

Compared to other additive manufacturing techniques, SLS allows the production of complex geometries without the need for support structures, as the surrounding unsintered powder provides mechanical stabilization during printing. The technology offers high dimensional accuracy (typically ±0.2%) and allows for controlled porosity depending on processing parameters. No chemical post-processing is required to achieve the desired mechanical properties, and excess powder can be mechanically removed and partially reused [31]. Together, these features make SLS a robust and reliable manufacturing approach for biomedical polymer components [27,30].

The selective laser sintering (SLS) manufacturing process consists of several sequential steps, including digital model preparation, manufacturing itself, and post-processing of the product [27,28]. The individual phases of the process significantly affect the quality, accuracy, and mechanical properties of the final product [32].

The manufacturing process was divided into the following steps.

1. Model preparation in software: The process begins with importing a 3D model in STL format into the appropriate software, where the model is placed in the workspace (build volume). In this phase, the orientation of the model and its placement in the print chamber are optimized to maximize production efficiency. At the same time, printing parameters can be adjusted, which affect the quality of the final product.
2. Preparing the printing unit: Before starting printing, it is necessary to prepare a container with powder material. Usually, a mixture of recycled and new powder is used.
3. Printing process: The production itself takes place by applying thin layers of powder, which are then selectively sintered with a laser beam according to the geometry of the model. After one layer is sintered, another layer of powder is applied and the process is repeated until the entire part is created.
4. Cooling the prints: After printing is finished, it is necessary to let the build chamber with the prints cool down (at least 24 h after completion). This step is key to minimizing internal stresses and deformations of the printed parts.
5. Removing excess powder: After cooling, the prints are removed from the powder bed and cleaned of unsintered material. Excess powder is collected and can be reused.
6. Final processing: The final step is the additional processing of the product, which mainly includes mechanical cleaning of the surface, for example by sandblasting. This process removes powder residues and improves the surface properties of the product. If necessary, additional treatments, can also be applied.

#### 2.2.1. Material PA12

Medical-grade polyamide 12 (PA12), commonly referred to as nylon 12, is one of the most commonly used materials in biomedical 3D printing [34]. This polymer is chemically inert, exhibits minimal immunogenicity, and rarely induces allergic reactions. It is considered a low-toxicity material that does not release harmful substances or decompose into toxic byproducts that could adversely affect biological tissues or cells. PA12 is compatible with standard sterilization techniques including gamma irradiation, ethylene oxide sterilization, and autoclaving without significant loss of material integrity. Compared to other 3D printing methods for processing PA12, powder bed methods such as MJF offer a significant advantage by enabling the production of geometrically complex components in any orientation without the need for support structures [34,35,36,37,38].

Polyamide 12 has been tested for USP Class I–VI [36,38], which includes irritation, acute systemic toxicity, and implantation, as well as cytotoxicity (according to Cytotoxicity—ISO 10993-5, Biological evaluation of medical devices—part 5: Tests for in vitro cytotoxicity [39]) and sensitization (following Sensitization—ISO 10993-10, Biological evaluation of medical devices—Part 10: Tests for irritation and skin sensitization [39]). It also meets the requirements of USP Class I–VI and the US Food and Drug Administration (FDA) guidelines for Intact Skin Surface Devices [34,35,36,37,38]. All technical parameters can be seen in a table below (Table 2).

#### 2.2.2. Construction of LED Cassettes

**Figure 4:**
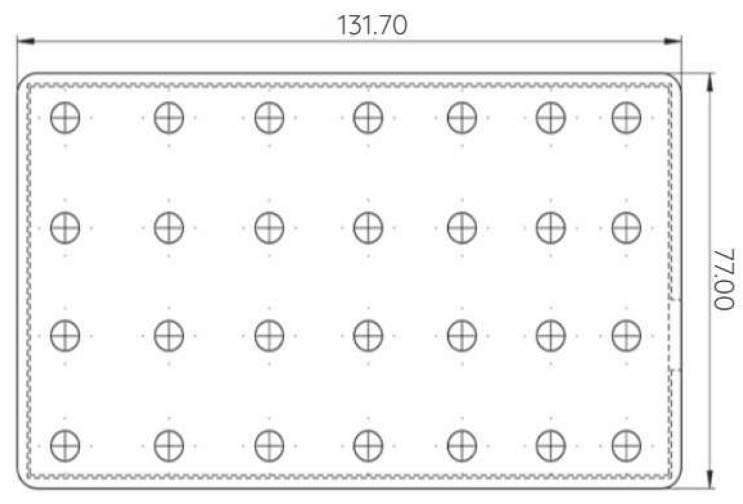
LED cassette dimensions [mm].

LED cassettes were designed as modular radiation sources with a defined wavelength. The mechanical part of the cassettes was realized using additive manufacturing technology (3D printing) from the thermoplastic material PETG, while the support plates contained a precisely defined spatial opening of the holes ensuring reproducible positioning of the individual discs (Figure 4).

The LEDs (OptoSupply, Hong Kong, China) were then mounted on a universal perforated board (a prototype printed circuit board without predefined conductive paths), which served as a mechanical carrier and at the same time as part of the electrical connections. The electrical connection was realized on the back of the board using soldered wires and tin connections, while the individual LEDs were arranged in series-parallel branches. The connection allows for effective distribution of the supply voltage and ensures stabilization of the current ratios in the individual branches, which is necessary to achieve uniform luminescence of the entire active cassette.

**Figure 5:**
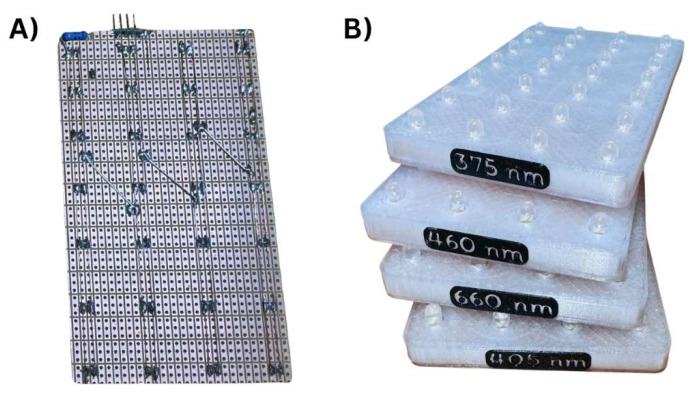
LED cassette: (**A**) open structure, (**B**) assembly in PETG block.

**Figure 6:**
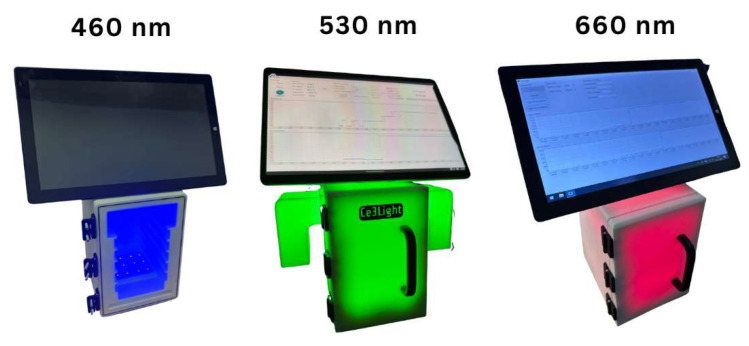
Ce3Light with different wavelength settings (460, nm 530 nm, 660 nm).

The finally assembled LED modules were integrated into printed components made of PETG, which ensure mechanical fixation and at the same time protect the electronic components (Figure 5). Thanks to this design, it is possible to achieve uniform irradiation of the workspace within the irradiation booth, which is crucial for ensuring the reproducibility of experimental conditions (Figure 6).

### 2.3. Software

**Figure 7:**
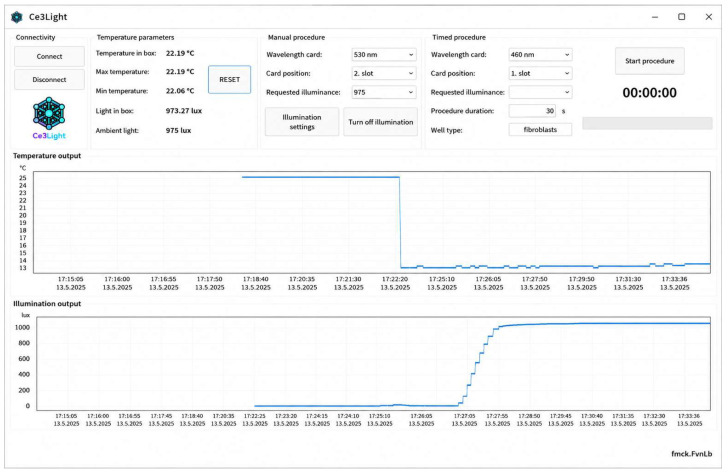
Ce3Light software.

For the precise control of experimental conditions, we developed a specialized software for Ce3Light (Figure 7), which serves as a central interface for communication with the irradiation device. The application architecture is designed to integrate data from multiple sensors in real time.

The main task of the software is to continuously monitor the temperature in the chamber and the illumination intensity, with both of these variables being recorded at second intervals. Data visualization is performed using two independent graphic modules, which allow the operator to retrospectively verify the stability of the environment, which is especially crucial during long-term irradiation of cell cultures.

The experiment setup process in the software is performed by defining the wavelength of the inserted LED card, for example 460 nm or 530 nm, and manually or automatically determining the target illumination intensity in lux. The system has intelligent feedback that is able to smoothly regulate the ramp-up of the light source to the desired value, thereby preventing unwanted light shocks. In addition to the light intensity, the software also closely monitors the temperature in the box, recording the maximum and minimum measured values since the start of irradiation.

In terms of the user interface, the software is divided into several functional blocks that allow for comprehensive experiment management. In the upper left part there is a connectivity and monitoring module for basic parameters, where the system displays in real time not only the current temperature and light intensity in the box, but also their extreme values (minimum and maximum) recorded during the ongoing session. This section serves for quick diagnostics of the stability of the environment before the irradiation procedure is started.

The central part of the control panel is reserved for defining manual and timed procedures. Here the user specifies the parameters of the inserted hardware, namely the wavelength of the LED card and its geometric position in the device. In addition to setting the target illumination intensity, the software in this section also allows metadata to be assigned to the experiment, such as the type of cultured cells and the exact duration of exposure in seconds. The upper right segment then contains the controls for starting the procedure along with a digital timer that provides visual feedback on the remaining irradiation time. The lower half of the interface is predominantly dedicated to the graphical analysis of the experiment. Two parallel graphs with a time axis record the development of temperature and illumination intensity. From these records, it is possible to identify the dynamics of the system, for example, the gradual increase in light output to the desired level or the stabilization of thermal equilibrium after activation of the light sources.

### 2.4. Validation of Ce3Light

The experimental part of the study was focused on validating the functional properties and operational stability of the Ce3Light irradiation box. The testing included the calibration of integrated sensors—the LUX DFR0026 luxmeter (DFRobot, Shanghai, China) and the LM35 thermometer (Texas Instruments, Dallas, TX, USA), which monitor the internal temperature and light intensity in real time during the experiments. The device was validated not only in laboratory conditions, but also during operation in a closed CO_2_ incubator. Following activation of the LED modules, the illumination intensity stabilized within approximately 8–12 s due to automatic regulation of the LED driving current. Subsequently, the internal temperature increased gradually until reaching a stable operating value. All biological experiments were initiated only after the illumination intensity had stabilized, ensuring reproducible irradiation conditions [43].

Cell culture and treatment

The human fibroblastic MRC-5 cell line was purchased from the American Type Culture Collection (ATCC No. CCL-171, Manassas, VA, USA). The cells were cultured in Modified Eagle’s Medium (MEM) without phenol red, supplemented with 10% (*v*/*v*) fetal bovine serum (Gibco, Grand Island, NY, USA), 1% non-essential amino acids (Gibco, USA), 2 mM glutamine (Gibco, USA) and 50 μg/mL penicillin–streptomycin solution (Gibco, USA). Cells were maintained at 37 °C in a sterile, humidified atmosphere containing 5% CO_2_. All the experiments were performed using MRC-5 cells in passages 5–15. MRC-5 cells were tested for mycoplasma contamination using the MycoAlert Mycoplasma Detection Kit (Lonza, Basel, Switzerland). All cells used in the experiments were mycoplasma-free.

MRC-5 cells were seeded in 100 µL of cell culture medium in 96-well plates at a density of 15 × 10^3^ cells per well for 24 h. The volume of 100 µL of culture medium corresponded to a height of 3.1 mm in each well. A 96-well plate (Nunc™ MicroWell™ 96-Well, Polystyrene, Nunclon Delta-Treated, Flat-Bottom Microplate; Thermo Scientific™, Waltham, MA, USA) was used for all experiments with the Ce3Light irradiation device. Illumination was performed through the bottom of the wells using interchangeable LED modules. The plate was positioned as close as possible to the LED modules, with a distance of approximately 50 mm. Cells were irradiated with monochromatic light at peak wavelengths of 460, 530, 660, or 800 nm for 24 h. For the 660 and 800 nm groups, an additional irradiation protocol consisting of 2 h of light exposure followed by 22 h of incubation under standard culture conditions was also evaluated. The technical specifications of the LED modules and the estimated irradiation parameters are summarized in Table 3. To estimate the effect of the irradiation, MRC-5 cells were also incubated without irradiation for 24 h (=control, non-irradiated). After 24 h, dehydrogenase activity and intracellular glutathione levels were measured in cells.

Glutathione assay

The glutathione (GSH) levels were measured using an optimized monochlorobimane assay [24] detecting glutathione in the cells. The working solution of monochlorobimane (MCB, Merck, Rahway, NJ, USA) was freshly prepared at the time of analysis by dilution in DPBS (pH 7.0; Merck, USA) and tempered at 37 °C for 30 min. 20 μL of MCB solution was added to the cells in 96-well plates and the measurement started immediately (final concentration of MCB in a well was 40 µM). The fluorescence intensity (Ex/Em = 394/490 nm) was measured kinetically for 10 min using a SPARK microplate reader (Tecan, Grödig, Austria). The fluorescence was expressed as the slope of a fluorescence change over time. The GSH levels were expressed as the percentage relative to GSH levels of control cells not exposed to the tested wavelengths of visible light (control = 100%).

Dehydrogenase activity assay

Dehydrogenase activity was determined using the resazurin assay which reflects the activity of both intra- and extramitochondrial dehydrogenases. The resazurin working solution (Merck, USA) was prepared by dissolving resazurin in DPBS 1× (pH 7.0; Merck, USA) to obtain a final concentration of 0.3 mg/mL. After cell treatment, 20 μL of the resazurin solution was added to cells cultured in 96-well plates, and fluorescence measurement was initiated immediately. Fluorescence intensity (Ex/Em = 530/590 nm) was measured kinetically for 20 min using a SPARK microplate reader (Tecan, Grödig, Austria) during incubation at 37 °C. Dehydrogenase activity was expressed as the percentage of total cellular dehydrogenase activity relative to control cells not exposed to the tested wavelengths of visible light (control = 100%).

Fluorescence Microscopy

Phalloidin FITC (Gibco, USA) staining of actin filaments was performed in MRC-5 cells. Cells were cultured in 200 μL of cell culture medium in cell culture chamber slides at a density of 15 × 10^3^ cells per well. After the treatment, cells were fixed with 3.7% (*v*/*v*) formaldehyde for 5 min at 37 °C. The cells were then permeabilized with 0.1% (*v*/*v*) Triton × 100 for 15 min at 37 °C. Subsequently, cells were incubated with phalloidin FITC (1 μM) for 30 min at 37 °C to visualize actin filaments. After 30 min of phalloidin FITC loading, 10 μL of Hoechst 33258 solution was added to the cells. The final concentration of Hoechst 33258 in a well was 2 μg × mL^−1^. After incubation, cells were washed two times with DPBS (pH 7.0; 1 mM; 37 °C). Actin filaments, protein expression (FITC filter, λEx/Em = 480/30 nm), and cell nuclei (DAPI filter, λEx/Em = 375/28 nm) were visualized with a Nikon Eclipse 80i fluorescence microscope (Nikon, Tokyo, Japan).

Statistical Analysis

All experiments were repeated two times independently. All measurements were performed in multiplets (n = 70–80). The results are expressed as (mean ± SD). Statistical analysis was performed using OriginPro 9.0.0 (OriginLab, Northampton, MA, USA). Statistical significance was analyzed using one way analysis of variance (ANOVA) followed by post hoc Tukey’s test to compare the results at the significance level *p* = 0.05.

## 3. Results

In this study, we aimed to evaluate the effect of visible (460, 530, 660 nm) or near-infrared light (800 nm) on cultivation of MRC-5 cells. The cells were irradiated at 460, 530, 660 or 800 nm for 24 h. As controls, MRC-5 cells were incubated without irradiation in the incubator for 24 h. During the testing of the effect of irradiation on MRC-5 cells, we observed an increase of temperature in the Ce3Light instrument placed in the incubator in comparison to the standard incubation temperature of 37 °C. The increase of temperature was associated with slight evaporation of the culture medium (<5 μL). The evaporation of the medium was uniform in all tested wells. The difference among maximal temperatures observed during irradiation of the cells was 0.5 °C, whereas the highest temperature 39.5 °C was measured at wavelength 530 nm (Table 4). The average temperature 38 ± 1 °C measured by the Ce3Light instrument was the same at all tested wavelengths.

**Figure 8:**
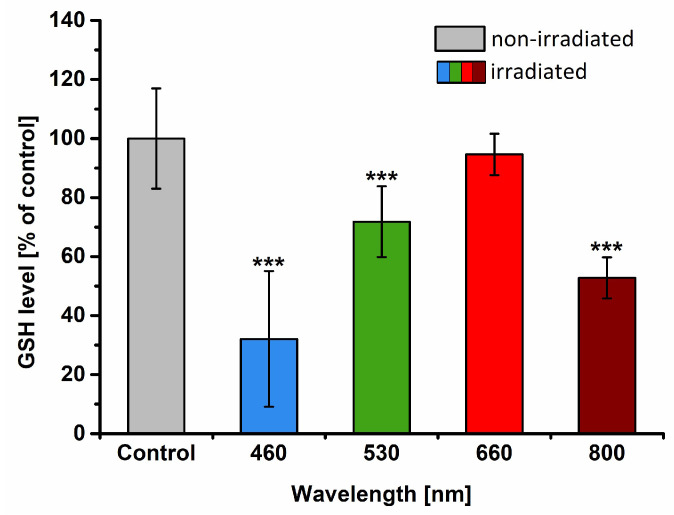
Intracellular glutathione levels. MRC-5 cells were irradiated at wavelengths 460/530/660/800 nm for 24 h. Non-irradiated were used as control cells. Glutathione levels were measured using monochlorobimane assay (Ex/Em = 394/490 nm). The data are presented as mean ±  SD (n = 70–80; at least two independent experiments). Statistical significance was analyzed using ANOVA followed by post hoc Tukey’s test to compare the results (***, *p* < 0.001, vs. non-irradiated cells).

The Ce3Light device was used to irradiate MRC-5 cells at tested wavelengths (460/530/660/800 nm) for 24 h. T_Avg_—The average temperature during the exposure. T_Max_—The maximal temperature during the exposure. To evaluate the functional state of both irradiated and non-irradiated MRC-5 cells, we measured intracellular glutathione levels [24] and dehydrogenase activity [44,45]. After 24 h, wavelengths 460 nm, 530 nm and 800 nm caused significant depletion of GSH levels (Figure 8). The largest decrease of GSH concentration by 68% (*p* < 0.001) occurred in cells cultured at 460 nm, compared to control cells. After 530 and 800 nm irradiation for 24 h, the decrease of GSH concentration in MRC-5 cells was by 28% (*p* < 0.001) and 47% (*p* < 0.001), respectively, compared to non-irradiated cells.

**Figure 9:**
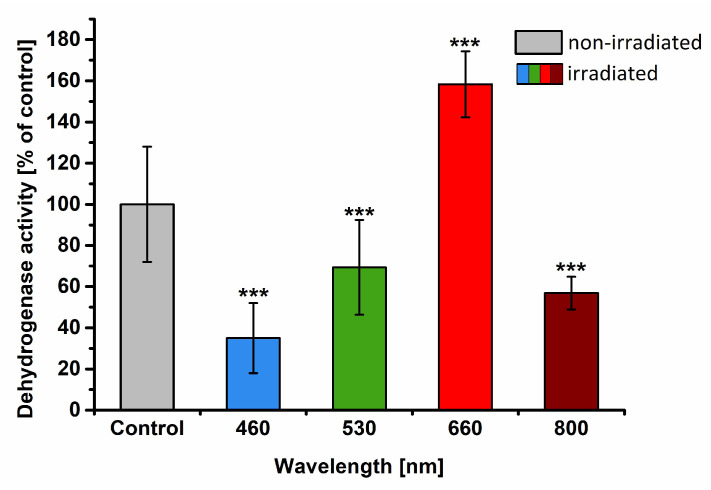
Intracellular dehydrogenase activity. MRC-5 cells were irradiated at wavelengths 460/530/660/800 nm for 24 h. Non-irradiated were used as control cells. Intracellular dehydrogenase activity was measured using resazurin assay (Ex/Em = 530/590 nm). The data are presented as mean  ±  SD (n = 70–80; at least two independent experiments). Statistical significance was analyzed using ANOVA followed by post hoc Tukey’s test to compare the results (***, *p* < 0.001, vs. non-irradiated cells).

According to the results from measurement of dehydrogenase activity, all tested wavelengths caused significant changes in comparison to non-irradiated cells (Figure 9). The largest decrease of dehydrogenase activity by 65% (*p* < 0.001) occurred in cells cultured at 460 nm, compared to controls. Moderate decreases of dehydrogenase activity by 43% and 31% (both *p* < 0.001) were detected in cells cultured at 530 and 800 nm, respectively, compared to non-irradiated cells. In contrast, during the testing of the effect of red wavelength (660 nm), the significant increase in dehydrogenase activity to 158 ± 16% (*p* < 0.001) occurred in MRC-5 cells compared to non-irradiated cells.

**Figure 10:**
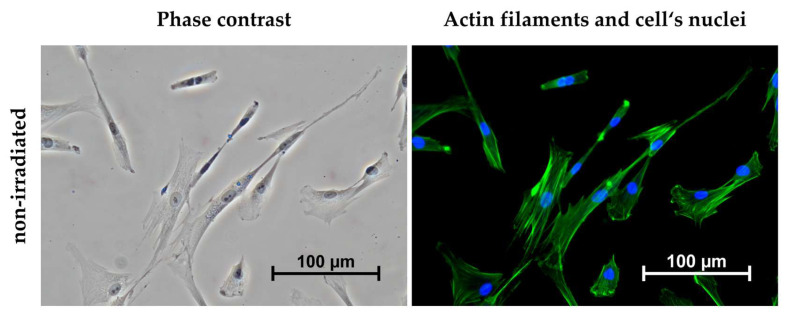
Photomicrographs of non-irradiated MRC-5 cells. The actin filaments were stained with the phalloidin-FITC probe (green; FITC filter, 480/30 nm), and the cell′s nuclei were stained with the Hoechst 33258 probe (blue; DAPI filter, 375/28 nm).

In addition to biochemical assays, we observed the morphology of MRC-5 cells using phase contrast and fluorescence microscopy. The actin filaments were stained with the phalloidin-FITC probe, and the cell′s nuclei were stained with the Hoechst 33258 probe. A preserved typical fibroblast morphology was observed in all visualized cells (Figure 10).

## 4. Discussion

The experimental validation of the Ce3Light system demonstrated that cellular responses to light stimulation were strongly dependent on the applied wavelength. Significant changes in both intracellular glutathione levels and dehydrogenase activity confirmed that photobiomodulation affects not only cellular metabolism but also redox homeostasis in MRC-5 fibroblasts. Our findings are consistent with previous studies reporting wavelength-dependent biological responses in fibroblasts and mesenchymal stem cells.

The most pronounced stimulatory effect was observed at 660 nm, where an increase in dehydrogenase activity indicated enhanced metabolic activity of the cells. Similar observations have been reported in human gingival fibroblasts (HGF), human adipose-derived stem cells (hASC), and L929 fibroblasts, where red light promoted proliferation, mitochondrial activity, and ATP production [13,14,15,18,20]. The beneficial effects of red light are commonly attributed to photon absorption by cytochrome c oxidase, resulting in increased mitochondrial respiration and activation of intracellular signaling pathways associated with cell survival and proliferation.

In contrast, exposure to blue light (460 nm) resulted in the largest decrease in intracellular glutathione levels and a reduction in metabolic activity. This observation could be attributed to an increase of intracellular ROS production and induction of oxidative stress observed in reports using short-wavelength light. Wang et al. demonstrated that blue light (415–420 nm) inhibited proliferation of human adipose-derived stem cells while simultaneously increasing ROS formation and intracellular calcium signaling [18,19]. Similarly, irradiation of keratinocytes, dermal fibroblasts and endothelial cells at 450 nm produced a dose-dependent response, where lower doses stimulated cellular activity while higher doses inhibited proliferation [23]. The marked depletion of glutathione observed in the present study therefore likely reflects an oxidative challenge induced by prolonged exposure to blue light. This observation is in agreement with the general trend reported across the visible spectrum, where the biological toxicity of light increases with decreasing wavelength. Similar findings were reported by Tonolli et al. [46] in HaCaT cells, while Ramakrishnan et al. [47] described cytotoxic effects of violet light in OST-5 cells. The higher photon energy associated with short wavelengths may contribute to increased oxidative stress and cellular damage.

Unexpectedly, irradiation at 530 nm also resulted in a decrease in both intracellular glutathione levels and dehydrogenase activity. Based on previous observations, green light is often considered biologically neutral or less effective than red and near-infrared wavelengths. However, Kim et al. [48] demonstrated that the cellular response to green light is highly dose-dependent, suggesting that the inhibitory effects observed in the present study may be associated with the specific irradiation conditions applied.

Interestingly, irradiation at 800 nm did not produce the stimulatory response frequently reported for near-infrared wavelengths. Previous studies described enhanced proliferation, differentiation and mitochondrial activity following exposure to wavelengths between 810 and 850 nm in hASCs [18], hiPSCs [49] and L929 fibroblasts [20]. The absence of a stimulatory effect at 800 nm may also be associated with the substantially lower irradiance delivered by the NIR LEDs compared to the visible wavelengths used in our study. Similar reductions in cellular viability following near-infrared irradiation have also been reported by Shimizu et al. [50], indicating that the biological effects of NIR light remain highly dependent on irradiation parameters and cellular context.

The present results also support the concept of a biphasic dose response, which has been repeatedly described in the PBM literature. Several authors observed that lower irradiation doses stimulated proliferation and differentiation, whereas excessive doses resulted in growth inhibition or cytotoxic effects. Such observations highlight the importance of precise control of irradiation conditions and demonstrate that biological outcomes cannot be predicted solely based on wavelength.

From a technological perspective, the study identified temperature regulation as an important factor influencing experimental reproducibility. During long-term irradiation, the temperature inside the system increased above standard cell culture conditions, reaching a maximum of 39.5 °C. Although no critical overheating occurred, even small temperature deviations may affect cellular metabolism and contribute to experimental variability. Consequently, active ventilation was incorporated into the next design iteration to improve thermal stability. This observation illustrates that the development of photobiomodulation platforms should not be considered solely an optical challenge but also a thermal and engineering one. Another advantage of the Ce3Light system is the possibility of placing the entire device inside a standard cell culture incubator with a controlled atmosphere of 5% CO_2_. This arrangement ensures that irradiated and control samples are maintained under identical environmental conditions, thereby minimizing the influence of external factors on experimental outcomes. A similar approach was described by Rosenberg et al. in 2020 [51], although in their study the light source was positioned above a 24-well culture plate. The use of defined wavelengths for cellular irradiation is also consistent with previous photobiomodulation studies, including those reported by Rossi et al. [52] and Kocherova et al. [53], further supporting the relevance of the experimental design adopted in the present work.

A major advantage of the Ce3Light system is its modular architecture and the use of additive manufacturing. The PA12-based 3D-printed construction enabled rapid modification of individual components and facilitated the implementation of design improvements during system development. In particular, the modular design allowed integration of active cooling elements without substantial reconstruction of the device. Such flexibility represents an important advantage compared with many custom-built irradiation systems reported in the literature.

Another important design decision was the use of LED technology instead of laser sources. Previous studies have demonstrated that many biological effects of photobiomodulation depend primarily on wavelength, irradiance, fluence and exposure time rather than on the coherence of the light source itself. The use of LED arrays therefore offers a cost-effective alternative while simultaneously providing more homogeneous illumination of multiwell plates. This feature is particularly important for in vitro experiments, where irradiation uniformity directly affects the reproducibility of biological responses. Although direct spatial mapping of irradiance across the 96-well plate was not performed in this study, the consistency of cellular responses across multiple wells suggests that the irradiation field was sufficiently homogeneous for biological validation. No systematic differences between peripheral and central wells were observed. Future work will include direct optical mapping of irradiance distribution across the entire plate area.

Overall, the results suggest that Ce3Light provides a suitable platform for controlled in vitro photobiomodulation experiments. At the same time, the study highlights the need for further optimization and standardization of irradiation parameters, which remain one of the principal limitations preventing direct comparison of PBM studies across different laboratories.

## 5. Conclusions

In this study, we designed, constructed, and experimentally validated Ce3Light, a modular LED-based irradiation platform for controlled in vitro photobiomodulation experiments. The system enabled stable and reproducible irradiation under standard cell culture conditions and demonstrated its suitability for evaluating wavelength-dependent cellular responses. Its modular design, interchangeable LED modules, and integrated monitoring of illuminance and temperature provide a flexible platform that can be readily adapted to different experimental requirements. Future work will focus on further optimization of the system, including improved thermal regulation, additional irradiation wavelengths, and integration with three-dimensional cell cultures and bioreactors. Beyond photobiomodulation research, the versatility of Ce3Light may also support future applications in biosensing, light-responsive drug delivery, and bioimaging.

## Figures and Tables

**Table 1:** Ce3Light parameters.

Parameter	Value
Width	136.00 mm
Height	196.00 mm
Depth	163.00 mm
Air inlet and outlet	Through vents (65 × 35 mm) on both sides
Thermometer	LM35 (0–100 °C)
Lux meter	LUX DFR0026 (1–6000 lux)
Number of microtiter plate positions	5 positions
Number of cards in the device at one time	1 card
Model material	PA12
Card material	PETG

**Table 2:** Technical parameters of PA12 material [34,38].

Property	Value	Standard/Conditions	Reference
Powder melting point (DSC)	187 °C	ASTM D3418	[40]
Melting temperature	180 °C	-	
Particle density	1.01 g/cm^3^	ASTM D792	[41]
Bulk density	0.425 g/cm^3^	ASTM D1895	[42]
Average particle size	60 µm	ASTM D3451	[39]
Tensile strength	50 MPa	At 50 mm/min	
Tensile modulus	1800 MPa	At 1 mm/min	
Flexural modulus	1700 MPa	At 2 mm/min, 10 N	

**Table 3:** Technical parameters of LED components.

Parameter	460 nm	530 nm	660 nm	800 nm
LED manufacturer	OptoSupply	OptoSupply	OptoSupply	OptoSupply
Number of LEDs	28	28	28	28
LED arrangement	7 × 4	7 × 4	7 × 4	7 × 4
Peak wavelength [nm]	455	525	660	805
Forward current, IF [mA]	19	20	20	45
Radiant power per LED [mW]	22	35	25	36
Total radiant power of LED [mW]	616	980	700	1008
Beam angle [2θ½, °]	15	30	15	30
Illuminated area [cm^2^]	109	109	109	109
Estimated irradiance [mW/cm^2^]	5.65	8.99	6.42	9.25
Exposure time [h]	24	24	24	24
Estimated fluence [24 h, J/cm^2^]	488.6	776.7	554.7	799.2
Estimated fluence [2 h, J/cm^2^]	40.7	64.7	46.2	66.6

Technical specifications were obtained from the manufacturers’ datasheets. The radiant power of the 800 nm LED module was corrected from the nominal datasheet value of 40 mW at 50 mA to the experimental operating current of 45 mA. The irradiance and fluence values were calculated from the nominal radiant power specified in the manufacturers’ datasheets, assuming uniform illumination of the illuminated area. These parameters represent theoretical estimates and were not determined by direct radiometric measurements.

**Table 4:** Instrumental conditions of the experiment.

Wavelength [nm]	Illuminance [lx]	T_Avg_ [°C]	T_Max_ [°C]	Evaporation of Medium
460	690 ± 10	38 ± 1	39.0	<5%
530	700 ± 10	38 ± 1	39.5	<5%
660	700 ± 10	38 ± 1	39.1	<3%
800	90 ± 5	38 ± 1	39.2	<3%

## Data Availability

Data will be provided upon a reasonable request.
